# Impact of corticosteroid treatment on clinical outcomes of influenza-associated ARDS: a nationwide multicenter study

**DOI:** 10.1186/s13613-020-0642-4

**Published:** 2020-02-27

**Authors:** Ming-Ju Tsai, Kuang-Yao Yang, Ming-Cheng Chan, Kuo-Chin Kao, Hao-Chien Wang, Wann-Cherng Perng, Chieh-Liang Wu, Shinn-Jye Liang, Wen-Feng Fang, Jong-Rung Tsai, Wei-An Chang, Ying-Chun Chien, Wei-Chih Chen, Han-Chung Hu, Chiung-Yu Lin, Wen-Cheng Chao, Chau-Chyun Sheu, for Taiwan Severe Influenza Research Consortium (TSIRC) Investigators

**Affiliations:** 1 Division of Pulmonary and Critical Care Medicine, Department of Internal Medicine, Kaohsiung Medical University Hospital, Kaohsiung Medical University, No. 100, Tz-You 1st Road, Kaohsiung, 807 Taiwan; 2 Department of Internal Medicine, School of Medicine, College of Medicine, Kaohsiung Medical University, Kaohsiung, Taiwan; 3 Graduate Institute of Clinical Medicine, College of Medicine, Kaohsiung Medical University, Kaohsiung, Taiwan; 4 Department of Respiratory Therapy, College of Medicine, Kaohsiung Medical University, Kaohsiung, Taiwan; 5 Department of Chest Medicine, Taipei Veterans General Hospital, Taipei, Taiwan; 6 Institute of Emergency and Critical Care Medicine, School of Medicine, National Yang-Ming University, Taipei, Taiwan; 7 Division of Chest Medicine, Department of Internal Medicine, Taichung Veterans General Hospital, Taichung, Taiwan; 8 Central Taiwan University of Science and Technology, Taichung, Taiwan; 9 Tunghai University, Taichung, Taiwan; 10 Department of Thoracic Medicine, Chang Gung Memorial Hospital, Taoyuan, Taiwan; 11 Department of Respiratory Therapy, Chang-Gung University College of Medicine, Taoyuan, Taiwan; 12 Division of Chest Medicine, Department of Internal Medicine, National Taiwan University Hospital, Taipei, Taiwan; 13 Division of Pulmonary and Critical Care Medicine, Department of Internal Medicine, Tri-Service General Hospital, National Defense Medical Center, Taipei, Taiwan; 14 Center for Quality Management, Taichung Veterans General Hospital, Taichung, Taiwan; 15 Division of Pulmonary and Critical Care, Department of Internal Medicine, China Medical University Hospital, Taichung, Taiwan; 16 Division of Pulmonary and Critical Care Medicine, Department of Internal Medicine, Kaohsiung Chang Gung Memorial Hospital, Kaohsiung, Taiwan; 17 Department of Respiratory Care, Chang Gung University of Science and Technology, Chiayi, Taiwan; 18 Faculty of Medicine, School of Medicine, National Yang-Ming University, Taipei, Taiwan; 19 Department of Medical Research, Taichung Veterans General Hospital, Taichung, Taiwan

**Keywords:** Influenza, Steroid, Glucocorticoid, Acute respiratory distress syndrome, Mortality, Pneumonia

## Abstract

**Background:**

Corticosteroid treatment has been widely used in the treatment of septic shock, influenza, and ARDS, although some previous studies discourage its use in severe influenza patients. This multicenter retrospective cohort study conducted in the intensive care units (ICUs) of eight medical centers across Taiwan aims to determine the real-world status of corticosteroid treatment in patients with influenza-associated acute respiratory distress syndrome (ARDS) and its impact on clinical outcomes. Between October 2015 and March 2016, consecutive ICU patients with virology-proven influenza infections who fulfilled ARDS and received invasive mechanical ventilation were enrolled. The impact of early corticosteroid treatment (≥ 200 mg hydrocortisone equivalent dose within 3 days after ICU admission, determined by a sensitivity analysis) on hospital mortality (the primary outcome) was assessed by multivariable logistic regression analysis, and further confirmed in a propensity score-matched cohort.

**Results:**

Among the 241 patients with influenza-associated ARDS, 85 (35.3%) patients receiving early corticosteroid treatment had similar baseline characteristics, but a significantly higher hospital mortality rate than those without early corticosteroid treatment [43.5% (37/85) vs. 19.2% (30/156), *p *< 0.001]. Early corticosteroid treatment was independently associated with increased hospital mortality in overall patients [adjusted odds ratio (95% CI) = 5.02 (2.39–10.54), *p *< 0.001] and in all subgroups. Earlier treatment and higher dosing were associated with higher hospital mortality. Early corticosteroid treatment was associated with a significantly increased odds of subsequent bacteremia [adjusted odds ratio (95% CI) = 2.37 (1.01–5.56)]. The analyses using a propensity score-matched cohort showed consistent results.

**Conclusions:**

Early corticosteroid treatment was associated with a significantly increased hospital mortality in adult patients with influenza-associated ARDS. Earlier treatment and higher dosing were associated with higher hospital mortality. Clinicians should be cautious while using corticosteroid treatment in this patient group.

## Background

Severe influenza is usually associated with high viral load and hypercytokinemia [1–4]. Based on the theoretical benefit in modulating hypercytokinemia and the clinical benefits shown in septic shock, corticosteroids have sometimes been used in severe influenza and influenza-associated acute respiratory distress syndrome (ARDS) [5, 6]. An European retrospective analysis of 208 patients with ARDS related to 2009 pandemic influenza A (H1N1), with 39.9% of patients receiving corticosteroid treatment, refuted the beneficial effects of corticosteroids for patients with influenza-associated ARDS and even showed an increased mortality associated with early corticosteroid treatment (within 3 days of mechanical ventilation) [7]. Another study of 2009 pandemic influenza A (H1N1)-related critical illness in Canada, with 46.1% of patients received corticosteroids, also revealed higher hospital mortality, ventilator days, and intensive care unit (ICU) days in patients using corticosteroids, while the odds ratio of association between corticosteroid treatment and hospital mortality decreased from 1.85 in the multivariable model to 0.96 after adjusting for time-dependent between-group differences using marginal structural modeling [8]. A Chinese study of avian influenza A (H7N9) viral pneumonia revealed a significantly higher 60-day mortality in patients receiving corticosteroids, and high-dose corticosteroid treatment was significantly associated with higher 30-day and 60-day mortalities [9]. A systematic review and meta-analysis including 9 cohort studies and 14 case–control studies also showed that corticosteroid treatment was associated with higher mortality in patients with influenza A (H1N1) infection [10]. The secondary analysis of a Spanish multicenter prospective cohort study showed that corticosteroid therapy was associated with increased ICU mortality in patients with severe influenza [11]. Because corticosteroids might be more frequently and/or earlier used in sicker patients, a solid conclusion about the association between corticosteroid treatments and mortality in severe influenza could not be made.

Corticosteroids might theoretically dampen both inflammation and fibrosis, the cardinal mechanisms for lung injury and adverse outcomes in ARDS, but the use of corticosteroids in ARDS remained controversial despite being extensively evaluated in a plethora of studies [12, 13]. Earlier studies using high-dose corticosteroids showed no survival benefit, whereas using low-dose corticosteroids appeared to improve survival [14]. The timing also matters, while starting methylprednisolone more than 14 days after ARDS onset significantly increased mortality [15]. A systematic review and meta-analysis including 8 randomized controlled trials (RCTs) and 10 cohort studies revealed heterogeneity in effects of corticosteroids on ARDS, which might be influenced by the duration of outcome measures and the etiologies of ARDS [5]. This meta-analysis showed that corticosteroid treatment was not associated with better long-term outcomes in ARDS, and moreover, was associated with increased risk of mortality in influenza-related ARDS [5].

Although the role of corticosteroid treatment in ARDS and influenza has been challenged, this treatment is still frequently endorsed by clinicians for patients with influenza-associated ARDS [9, 13]. A large international multicenter prospective observational cohort study across 50 countries showed that 17.9% of ARDS patients received high-dose corticosteroids (≥ 1 mg/kg methylprednisolone equivalent dose) [16]. In a Chinese study of influenza pneumonia, 70.8% of patients received corticosteroids [9].

The aims of this study were to understand the real-world condition of corticosteroid treatment in influenza-associated ARDS, and to investigate the impact of corticosteroid treatment on clinical outcomes in patients with influenza-associated ARDS.

## Methods

### Study cohort

This multicenter retrospective cohort study was conducted in the ICUs in eight medical centers across Taiwan during the 2015–2016 influenza epidemic [17–19]. The study was approved by the Institutional Review Board in each participating hospital.

All patients admitted to the ICUs between October 1, 2015 and March 31, 2016 with a diagnosis of virology-proven influenza infections were screened, and patients fulfilled the ARDS criteria and received invasive mechanical ventilation were enrolled in the study. The diagnosis of influenza was confirmed by the Taiwan Centers for Disease Control based on the rapid influenza diagnostic test, reverse transcription-polymerase chain reaction, or viral culture. The diagnosis and grading of ARDS was determined according to the Berlin definition [20].

### Measures

Medical records of the eligible patients were reviewed and data were collected with a standardized case report form in the participating ICUs. The relevant information, including demographics, comorbidities, laboratory tests, influenza type, severity score, and specific treatments, were collected. The calendar day of ICU admission was defined as the first ICU day (ICU Day 1). The details of corticosteroid use (including the timing of initiation, dosing, and type of medications) were recorded, and the doses were converted to hydrocortisone equivalent doses. The cumulative corticosteroid dose was calculated from the ICU admission to the end of the specified day after ICU admission. Bacterial co-infections were defined as positive bacterial cultures from blood, pleural effusion, lower respiratory tract secretion, or urine samples within 48 h of ARDS diagnosis. Hospital mortality was taken as the primary outcome in this study.

### Statistical analysis

Data are presented in number (%) or median (25th–75th percentiles), as appropriate. Categorical variables and continuous variables were compared using *χ*^2^ test and Mann–Whitney *U* test, respectively. Survival times were estimated using the Kaplan–Meier method, with differences between the groups compared using log-rank test. Cox proportional hazards regression analysis was used to identify the effects of variables on survival. Logistic regression analysis was used to identify the effects of variables on hospital mortality. Variables with a *p* < 0.05 in univariate models were selected into the multivariable model, using a stepwise algorithm with criteria of *p* > 0.05 for eliminating variables. Missing values were replaced by the corresponding overall median values for multivariable analyses.

To account for residual confounding by indication of the associations between early corticosteroid treatment and clinical outcomes, variables potentially associated with early corticosteroid treatment, including age, sex, Acute Physiology and Chronic Health Evaluation (APACHE) II score, influenza type, ARDS severity, bacterial coinfection, vasopressor infusion, prone positioning, extracorporeal membrane oxygenation (ECMO) treatment, and chronic airway disease, were included in a logistic regression model with early corticosteroid treatment as the dependent variable to determine a propensity score for treatment. A propensity score-matched cohort was selected from the original cohort and similar analyses were performed to confirm our findings from the original cohort.

All statistical analyses were performed using SAS system (version 9.4 for Windows, SAS Institute Inc., Cary, NC, USA). The statistical significance level was set at a two-sided *p* value of < 0.05. A hazard ratio (HR) or an odds ratio (OR) was reported along with 95% confidence interval (CI).

## Results

### Analyses of the whole study population

Totally, 336 patients admitted to the ICUs with virology-proven complicated influenza were screened (Additional file 1: Fig. S1). After excluding 54 patients without ARDS, 35 patients who did not receive mechanical ventilation, and 6 patients with incomplete data, the data of the remaining 241 patients, including 174 (72.2%) survivors and 67 (27.8%) non-survivors, were used for analyses (Additional file 1: Table S1).

A substantial proportion of patients with influenza-associated ARDS in our study population had received corticosteroid treatment during their ICU stay. By the end of the third ICU day, 35.3% of patients had received ≥ 200 mg hydrocortisone equivalent dose, and the proportion increased to 57.7% by the end of the second week after ICU admission (Additional file 1: Fig. S2).

**Fig. 1:**
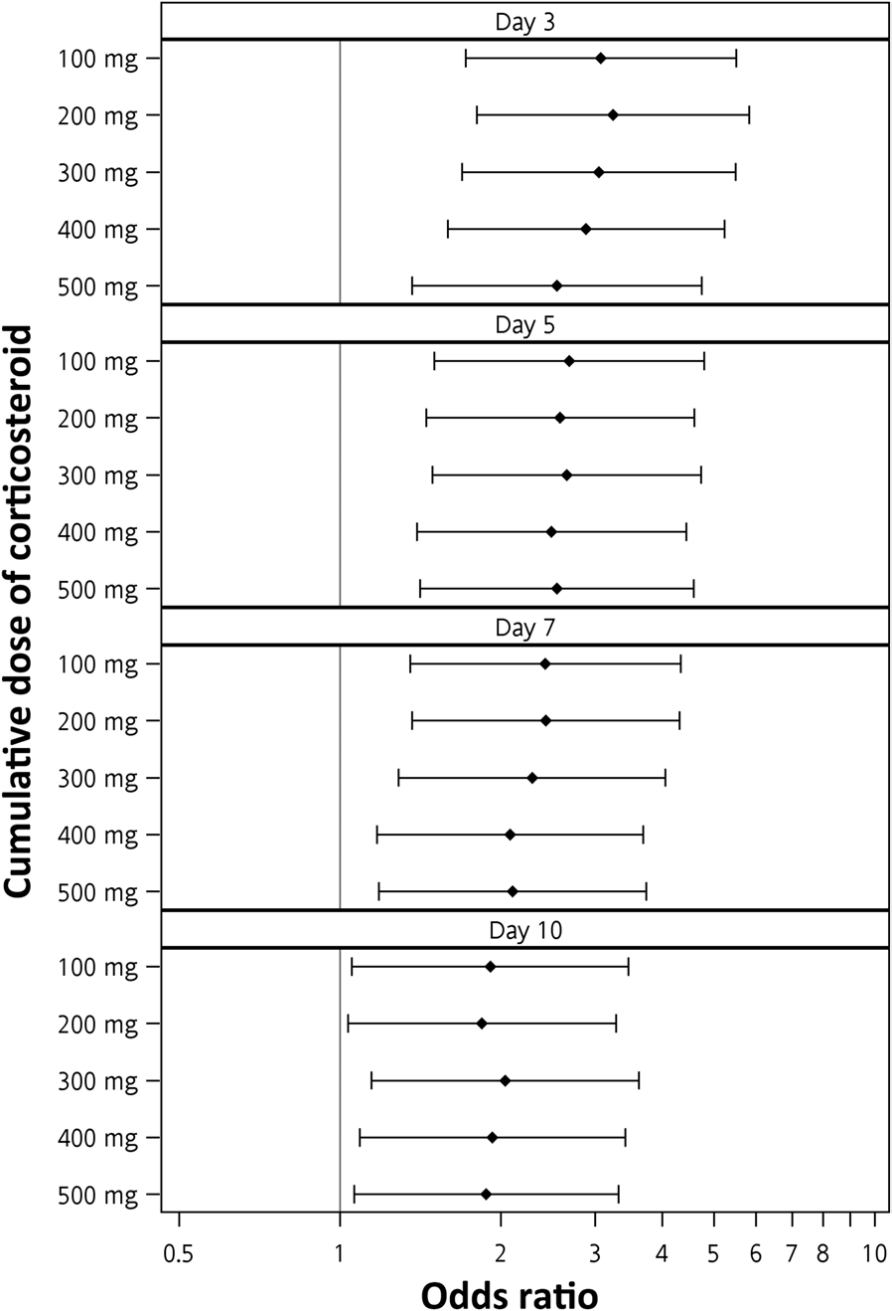
Sensitivity analysis. Using univariate logistic regression analyses, the effects of corticosteroid treatment (defined by various criteria) on the hospital mortality were assessed, and the odds ratios (with 95% confidence intervals) were presented. Corticosteroid treatment was defined by achieving various cumulative hydrocortisone equivalent doses by various ICU days. Corticosteroid treatment was significantly associated with hospital mortality for all proposed criteria. There was a trend that the earlier use of corticosteroid was associated with the higher odds of hospital mortality. The criteria using cumulative hydrocortisone equivalent dose of ≥ 200 mg by the end of the third ICU day (ICU Day 3) provided the highest odds of hospital mortality

We performed a sensitivity analysis, which used univariate logistic regression analyses to assess the effects of various cumulative doses of corticosteroids by various ICU days on the hospital mortality (Fig. 1). Corticosteroid treatment was significantly associated with hospital mortality for all proposed criteria, while there was a trend that the earlier use of corticosteroids was associated with the higher odds of hospital mortality in patients with influenza-associated ARDS. The criteria using cumulative hydrocortisone equivalent dose of ≥ 200 mg by the end of the third ICU day provided the highest odds of hospital mortality in the univariate logistic regression analysis, and was therefore used as the definition for “early corticosteroid treatment” in the subsequent analyses.

**Fig. 2:**
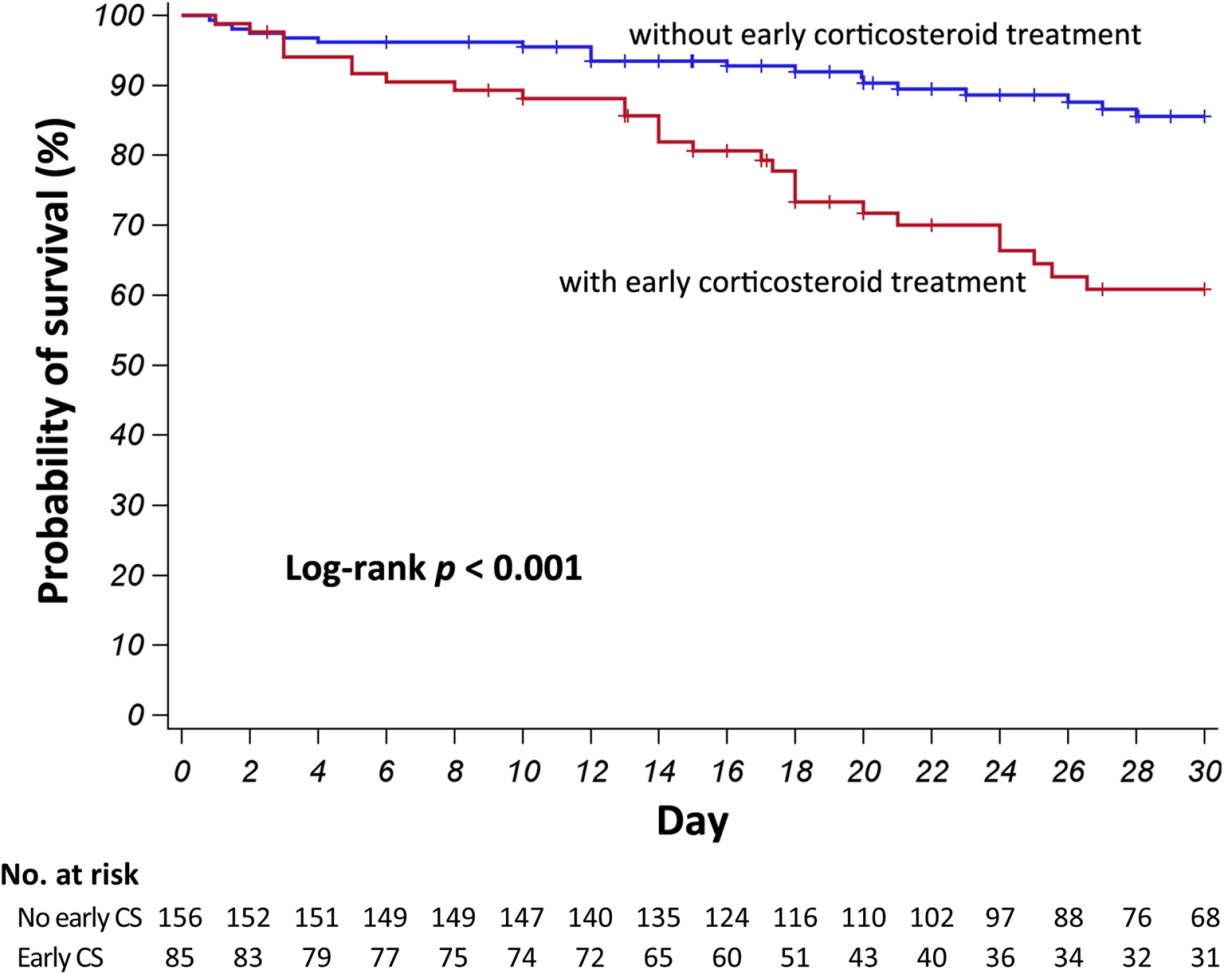
Probability of survival from ICU admission to Day 30. The patients were classified by whether they received early corticosteroid (CS) treatment or not

**Fig. 3:**
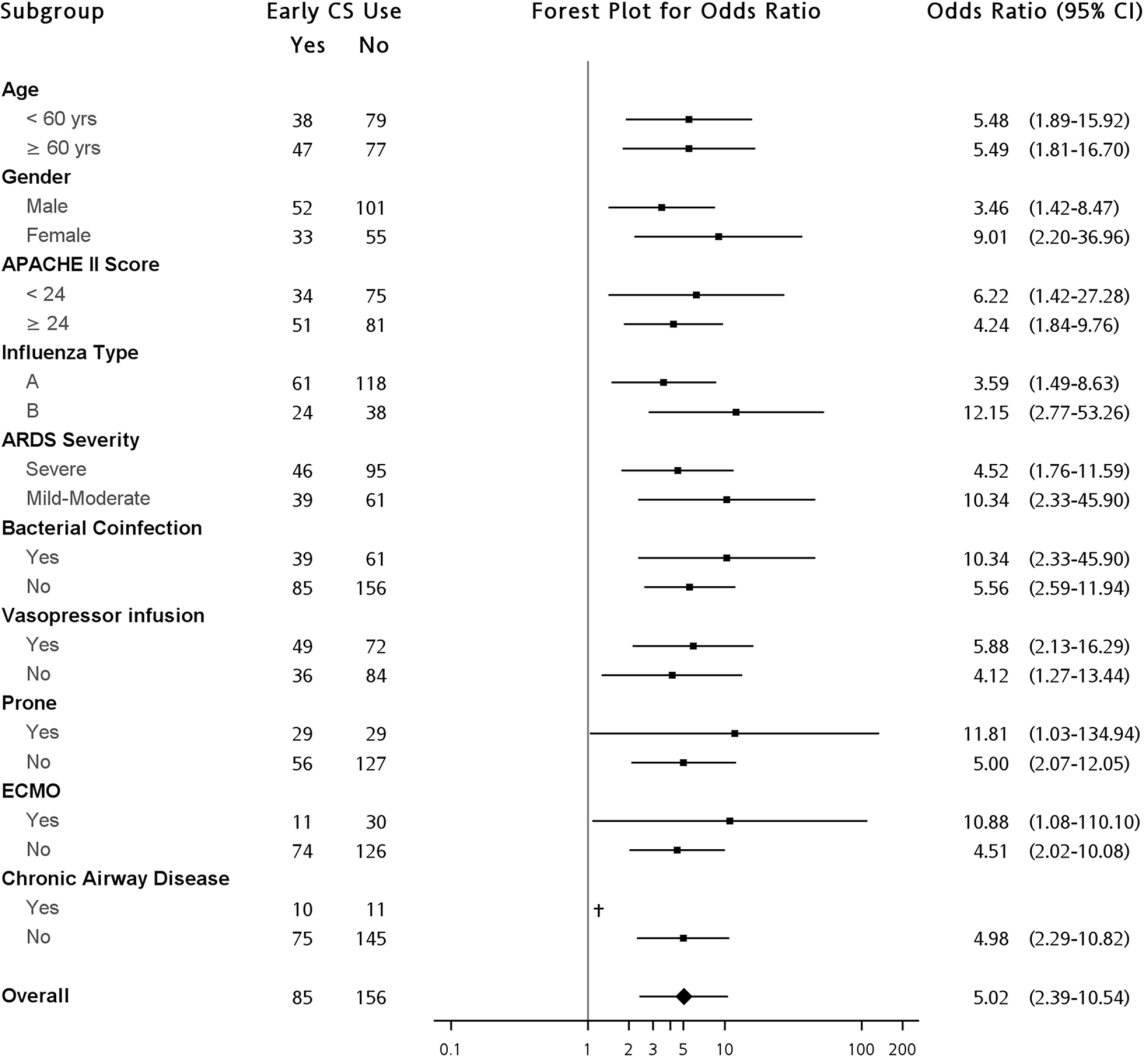
Forest plot of stratified analyses showing adjusted odds ratio of early corticosteroid treatment. The subgroups were classified by demographic and disease characteristics. Odds ratios for mortality were adjusted for APACHE II score, underlying malignancy, influenza type, and ECMO treatment, which were selected from the multivariable model in Table 2. ^†^ The adjusted OR could not be calculated due to small sample size

Using this definition, the study population was divided into 85 patients received early corticosteroid treatment and 156 patients without early corticosteroid treatment (Table 1). The patients received early corticosteroid treatment had higher hospital mortality rate than those without early corticosteroid treatment [43.5% (37/85) vs. 19.2% (30/156), *p* < 0.001]. The patients receiving early corticosteroid treatment also had lower probability of survival than those without early corticosteroid treatment (log rank *p* < 0.001) (Fig. 2). Multivariable logistic regression analysis constructed with a stepwise variable selection method showed that early corticosteroid treatment remained an independent risk factor for hospital mortality [adjusted OR (95% CI) = 5.02 (2.39–10.54), *p* < 0.001] after adjusting for APACHE II score, underlying malignancy, influenza type, and ECMO treatment (Table 2). On subgroup analyses, early corticosteroid treatment remained an independent risk factor for hospital mortality in nearly all subgroups (Fig. 3). In the Cox proportional hazards regression analysis adjusting for APACHE II score, underlying malignancy, influenza type, and ECMO treatment, early corticosteroid treatment remained an independent risk factor for mortality [adjusted HR (95% CI) = 2.49 (1.46–4.24), *p* < 0.001].

**Table 1:** Characteristics and outcomes between influenza-associated ARDS patients with versus without early corticosteroid treatment

Variables	Early CS treatment (N = 85)	No early CS treatment (N = 156)	*p* value
Age	61.0 (56.0–68.0)	59.0 (49.0–65.5)	0.211
Male gender	52 (61.2)	101 (64.7)	0.674
APACHE II score	24.0 (18.0–30.0)	23.0 (17.0–29.0)	0.129
BMI	24.6 (22.1–28.1)	24.8 (21.2–28.1)	0.808
Comorbidity			
Malignancy	7 (8.2)	22 (14.1)	0.217
Diabetes	26 (30.6)	43 (27.6)	0.655
Cerebrovascular disease	8 (9.4)	10 (6.4)	0.445
Chronic airway disease	10 (11.8)	11 (7.1)	0.237
End-stage renal disease	7 (8.2)	7 (4.5)	0.257
Congestive heart failure	11 (12.9)	15 (9.6)	0.515
Influenza			0.539
Type A	61 (71.8)	118 (75.6)	
Type B	24 (28.2)	38 (24.4)	
PaO_2_/FiO_2_ ratio	87.5 (56.0–142.3)	84.0 (62.0–149.6)	0.670
Severity of ARDS			0.539
Severe ARDS	46 (54.1)	95 (60.9)	
Moderate ARDS	32 (37.6)	41 (26.3)	
Mild ARDS	7 (8.2)	20 (12.8)	
Laboratory data			
WBC count, ×10^3^/μL	9.6 (5.5–15.5)	8.8 (5.9–13.0)	0.253
Hemoglobin, g/dL	12.5 (10.5–14.2)	11.9 (10.0–13.5)	0.102
Platelet count, ×10^3^/μL	150.0 (108.5–200.5)	149.0 (102.0–210.0)	0.770
Albumin, g/dL	2.9 (2.5–3.3)	2.9 (2.5–3.2)	0.670
C-reactive protein, mg/dL	14.1 (4.2–23.3)	14.9 (6.7–23.0)	0.727
Bacterial coinfections^a^	14 (16.5)	17 (10.9)	0.231
Specific treatment			
Mechanical ventilation	85 (100.0)	156 (100.0)	0.539
Prone positioning or ECMO	36 (42.4)	56 (35.9)	0.334
Prone positioning	29 (34.1)	29 (18.6)	*0.011*
ECMO	11 (12.9)	30 (19.2)	0.281
Vasopressor infusion	49 (57.6)	72 (46.2)	0.105
Hemodialysis	16 (18.8)	24 (15.4)	0.587
Clinical outcomes			
Hospital mortality	37 (43.5)	30 (19.2)	*< 0.001*
Hospital days in survivors	24.8 (17.1–40.1)	28.2 (19.1–44.4)	0.475
ICU days in survivors	13.8 (8.4–23.0)	14.8 (9.1–21.8)	0.664
Ventilator days in survivors	11.9 (8.4–25.3)	14.4 (7.8–21.7)	0.953
ICU-free days at Day 28	0.0 (0.0–15.2)	9.2 (0.0–17.6)	*0.012*
Ventilator-free days at Day 28	0.0 (0.0–17.1)	11.0 (0.0–19.6)	*0.009*

*APACHE II* Acute Physiology and Chronic Health Evaluation II, *ARDS* acute respiratory distress syndrome, *BMI* body mass index, *CS* corticosteroid, *ECMO* extracorporeal membrane oxygenation, *FiO*_*2*_ fraction of inspired oxygen, *PaO*_*2*_ partial pressure of arterial oxygen, WBC white blood cell

Statistics are presented as the median (25th–75th percentiles) for continuous variables and as number (%) for categorical variables, as appropriate. *p* values are calculated by Mann–Whitney *U* test (or known as Wilcoxon rank sum test) and Chi-square test for continuous and categorical variables, respectively

^a^Bacterial coinfection was defined as positive bacterial cultures from blood, pleural effusion, lower respiratory tract secretion, or urine samples within 48 h of ARDS diagnosis

**Table 2:** Univariate and multivariable analyses of factors associated with hospital mortality in patients with influenza-associated ARDS

Variables	Univariate analysis^a^		Multivariable analysis^†^	
	OR (95% CI)	*p* value	Adjusted OR (95% CI)	*p* value
APACHE II score	1.11 (1.07–1.16)	< 0.001	1.12 (1.07–1.17)	*< 0.001*
PaO_2_/FiO_2_ ratio	0.99 (0.99–1.00)	0.007		
WBC	1.00 (1.00–1.00)	0.015		
Albumin, g/dL	0.55 (0.31–0.97)	0.039		
Malignancy	2.80 (1.27–6.18)	0.011	2.71 (1.06–6.90)	0.037
Influenza type A	0.50 (0.27–0.93)	0.028	0.38 (0.18–0.82)	0.013
ECMO	4.53 (2.25–9.14)	< 0.001	8.51 (3.52–20.55)	*< 0.001*
Vasopressor infusion	2.89 (1.59–5.25)	0.001		
Hemodialysis	2.54 (1.26–5.12)	0.009		
Early CS treatment	3.24 (1.80–5.81)	< 0.001	5.02 (2.39–10.54)	*< 0.001*

*APACHE II* Acute Physiology and Chronic Health Evaluation II, *ARDS* acute respiratory distress syndrome, *BMI* body mass index, *CI* confidence interval, *CS* corticosteroid, *ECMO* extracorporeal membrane oxygenation, *FiO*_*2*_ fraction of inspired oxygen, *PaO*_*2*_ partial pressure of arterial oxygen, *OR* odds ratio, *WBC* white blood cell

^a^The variable representing early CS treatment, basic demographic variables, and all clinical variables possibly associated with hospital mortality were analyzed in univariate logistic regression models

^†^We replaced missing values (APACHE II score in 4 patients, WBC count in 2 patients, and albumin in 40 patients) by the corresponding overall median values for the multivariable regression analysis. Variables associated with hospital mortality with a *p* value < 0.05 in univariate models were selected into the multivariable logistic regression model, using a stepwise algorithm with criteria of *p* > 0.05 for eliminating variables

The multivariable logistic regression models were also used to investigate the effect of dosing and timing of corticosteroid treatment on hospital mortality. The odds of hospital mortality significantly increased by 9% (2–16%), 6% (2–11%), and 4% (1–8%) with every 100 mg of cumulative hydrocortisone equivalent dose within 3, 5, 7 days after ICU admission, respectively (Additional file 1: Table S2), suggesting that earlier corticosteroid treatment and higher dosing were associated with higher hospital mortality. As shown in Additional file 1: Table S3, receiving corticosteroid treatment to a cumulative hydrocortisone equivalent dose reaching ≥ 200 mg within 3 days after ICU admission increased the odds of hospital mortality [adjusted OR (95% CI) = 4.58 (2.06–10.17), *p* < 0.001].

Because corticosteroid treatment might predispose patients to bacterial infections, we further analyzed the associations between corticosteroid treatment and subsequent ICU-acquired bacterial infections, defined as positive culture results from the samples collected after the third ICU day. Early corticosteroid treatment was associated with a significantly increased odds of subsequent bacteremia [adjusted OR (95% CI) = 2.37 (1.01–5.56)] and a trend of increased odds of positive culture from any samples (Additional file 1: Table S4). However, the subsequent ICU-acquired bacteremia or positive culture results from any samples was not independently associated with increased odds of hospital mortality, whereas positive culture results from respiratory samples collected after the third ICU day was independently associated with increased odds of hospital mortality [adjusted OR (95% CI) = 2.20 (1.01–4.81), *p* = 0.048] (Additional file 1: Table S5).

In our study population, 154 patients (63.9%) had ever received corticosteroid treatment at any dose within 14 days after ICU admission, including 104 survivors and 50 non-survivors (Additional file 1: Table S6). Compared with the survivors, the non-survivors had significantly shorter interval between ICU admission and the start of any corticosteroid treatment (*p* = 0.036) and a trend for longer duration of corticosteroid treatment (*p* = 0.054), whereas the total dose of corticosteroid used by the 14th day after ICU admission was similar in the survivors and non-survivors.

### Analyses of the propensity score-matched cohort

To further confirm our findings, we built a propensity score-matched cohort, containing 85 patients with early corticosteroid treatment and 85 patients without early corticosteroid treatment (Additional file 1: Table S7). Both groups had similar clinical characteristics and received similar specific treatments. The patients with early corticosteroid treatment had a higher hospital mortality rate than those without early corticosteroid treatment [43.5% (37/85) vs. 14.1% (18/85), *p* < 0.001]. The patients with early corticosteroid treatment also had lower probability of survival than those without early corticosteroid treatment (log rank *p* = 0.004) (Additional file 1: Fig. S3). Multivariable logistic regression analysis constructed with a stepwise variable selection method showed that early corticosteroid treatment remained an independent risk factor for hospital mortality [adjusted OR (95% CI) = 4.30 (1.90–9.75), *p* < 0.001] after adjusting for APACHE II score and ECMO treatment (Additional file 1: Table S8). On subgroup analyses, early corticosteroid treatment remained an independent risk factor for hospital mortality in nearly all subgroups (Additional file 1: Fig. S4). In the Cox proportional hazards regression analysis adjusting for APACHE II score and ECMO treatment, early corticosteroid treatment remained an independent risk factor for mortality [adjusted HR (95% CI) = 2.62 (1.46–4.70), *p* = 0.001].

## Discussion

In this multicenter cohort study, we found that a substantial proportion of patients with influenza-associated ARDS had received corticosteroid treatment and more than a third of them received ≥ 200 mg hydrocortisone equivalent dose within 3 days after ICU admission, although some previous literatures did not support this treatment. Higher APACHE II score, underlying malignancy, influenza type B, ECMO, and early corticosteroid treatment were significantly associated with hospital mortality in patients with influenza-associated ARDS. The risk of hospital mortality increased with the earlier use of corticosteroid treatment and with the higher cumulative dose of corticosteroid treatment. The analyses of a propensity score-matched cohort showed consistent results.

During the 2009 pandemic period, the World Health Organization published revised guidelines for the pharmacological management of pandemic influenza A (H1N1) [21]. Although “patients who have severe or progressive clinical illness, including viral pneumonitis, respiratory failure, and ARDS due to influenza virus infection, should not be given systemic corticosteroids unless indicated for other reasons or as part of an approved research protocol” have been clearly stated, the adherence to this suggestion remained poor [2, 21]. In the Chinese study collecting 613 patients of avian influenza A (H7N9) viral pneumonia between April 2013 to March 2015, as much as 70.8% of patients received corticosteroids [9]. Similarly, in our study, 57.7% of patients had received ≥ 200 mg hydrocortisone equivalent dose within 2 weeks after ICU admission and 63.9% of patients had ever received corticosteroid treatment at any dose during this period.

Many studies have investigated the role of systemic adjunctive corticosteroid treatment in pneumonia. A systematic review and meta-analysis including 13 RCTs showed that systemic corticosteroid treatment significantly decreased the need for mechanical ventilation and the risk of incident ARDS in adult patients hospitalized with severe community-acquired pneumonia (CAP), while a significantly decreased mortality was only found in those with severe pneumonia [22]. A large double-blind, multicenter, randomized, placebo-controlled study also showed prednisone treatment for 7 days shortened the time to clinical stability in patients admitted with CAP [23].

The excessive production of pro-inflammatory cytokines in sepsis may lead to relative adrenal insufficiency and/or peripheral glucocorticoid resistance [24]. Corticosteroid treatment decreases pro-inflammatory mediators and improves innate immunity [25, 26]. A multicenter RCT showed that low-dose hydrocortisone and fludrocortisone significantly reduced the mortality in patients with septic shock [27]. Another multicenter RCT of septic shock patients showed that hydrocortisone treatment hastened the reversal of shock, but did not improve survival [28]. Two large RCTs, APROCCHSS and ADRENAL, both showed the effect of corticosteroid treatment in enhancing shock reversal and decreasing the duration of mechanical ventilation in patients with septic shock [29, 30]. APROCCHSS showed that hydrocortisone plus fludrocortisone treatment decreased the 90-day mortality, whereas ADRENAL showed no significant difference in 90-day mortality in patients receiving hydrocortisone or those receiving placebo [29, 30]. Our study showed that shock (vasopressor infusion) was associated with significantly increased hospital mortality. However, the need of vasopressor infusion was excluded during stepwise variable selection for composing the multivariable model, suggesting that corticosteroid treatment might be a greater predictor for hospital mortality. In addition, the results of subgroup analysis showed that early corticosteroid was associated with increased hospital mortality in both vasopressor group and no vasopressor group, suggesting this association was independent of septic shock.

The underlying mechanisms why corticosteroid treatment increased mortality in influenza-associated ARDS remained unclear. In a large RCT of septic shock, patients treated with hydrocortisone therapy had more episodes of superinfections than those receiving placebo [28]. However, the Canadian study of 2009 pandemic influenza A (H1N1)-related critical illness showed no significant difference in nosocomial (respiratory and bloodstream) infections between the patients receiving corticosteroids and the others [8]. Our current study showed that early corticosteroid treatment might increase the risk of subsequent bacteremia, whereas the isolation of bacteria from respiratory samples was associated with increased hospital mortality.

Our study has several strengths. Firstly, our study enrolled patients from multiple medical centers across Taiwan, providing a great opportunity to investigate the real-world circumstance. Designing and conducting an RCT for pandemic critical illness is never easy [31]. To the best of our knowledge, no RCT discussing the role of corticosteroid treatment in influenza-associated ARDS has been done, and our study is one of the largest cohort studies focusing on this topic to date. Secondly, we only included patients receiving invasive mechanical ventilation for influenza-associated ARDS. In contrast to the previous studies, which included either patients with milder influenza or patients with ARDS from various etiologies, our homogenous cohort provided us a better opportunity for investigating the effect of corticosteroid treatment in the patients with influenza-associated ARDS [1, 2, 5, 9–11]. Thirdly, we performed thorough analyses, using variable timing and dosing definitions for corticosteroid treatment. These analyses not only showed consistent results, but also showed the dose-dependent and timing-related effects of corticosteroid treatment.

Nonetheless, our study still has limitations. Firstly, the retrospective nature of our study resulted in few missing values despite the effort in data collection, and might also bring some bias, such as confounding by indications of corticosteroid treatment (i.e., corticosteroid treatment might be used more frequently and earlier in sicker patients). A hard outcome of hospital mortality was therefore adopted. In a Canadian study of influenza-associated critical illness, the use of corticosteroid treatment was mainly associated with hemodynamic instability and chronic airway disease [32]. The subgroup analyses, classifying patients by vasopressor infusion or chronic airway disease, showed consistent results in our study. We also performed multivariable analyses to adjust all possible confounders. To account for residual confounding by indication, we performed another set of analyses in a propensity score-matched sub-cohort, with increased comparability between groups, and found consistent results. Nevertheless, despite efforts to mitigate the effects of residual confounding factors, this potential problem could not be totally solved. The results of this study must therefore be interpreted with caution. Further RCTs or well-designed prospective studies are warranted to understand the benefits or harms associated with corticosteroid treatment in patients with influenza-associated ARDS. Secondly, the effects of different types of corticosteroids were not investigated in the current study because some patients used more than one type of corticosteroid and our study had insufficient power to investigate this aspect. To investigate the effect of corticosteroids, we transfer all corticosteroids into hydrocortisone equivalent dose. Although we believe our finding is a class effect of corticosteroids, the differences between various corticosteroids need to be investigated in a larger study in the future.

## Conclusions

The current study showed that early corticosteroid treatment was associated with a significantly higher hospital mortality in adult patients with influenza-associated ARDS. The earlier the treatment, the higher the mortality. The higher the corticosteroid dose, the higher the mortality. While further RCTs or well-designed prospective studies are warranted to confirm our findings, we suggest that clinicians should be cautious about using corticosteroid treatment in this patient group.

## Supplementary information

**Additional file 1.** Additional tables and figures.

## Data Availability

Not applicable.

## Abbreviations

APACHE II: Acute Physiology and Chronic Health Evaluation II
ARDS: Acute respiratory distress syndrome
BMI: Body mass index
CI: Confidence interval
CS: Corticosteroid
ECMO: Extracorporeal membrane oxygenation
FiO_2_: Fraction of inspired oxygen
HR: Hazard ratio
ICU: Intensive care unit
OR: Odds ratio
PaO_2_: Partial pressure of arterial oxygen
RCT: Randomized controlled study
WBC: White blood cell
