# Supplementary material for: Impact of corticosteroid treatment on clinical outcomes of influenza-associated ARDS: a nationwide multicenter study
Source: Ann Intensive Care. 2020 Feb 27;10:26. doi: 10.1186/s13613-020-0642-4 (PMC7046839; doi:10.1186/s13613-020-0642-4)
Supplement: Supplementary file 1 — Additional file 1. Additional tables and figures. [file 13613_2020_642_MOESM1_ESM.docx]

**Additional Information**

**for**

**Impact of corticosteroid treatment on clinical outcomes of
influenza-associated ARDS: a nationwide multicenter study**

**Authors:**

Ming-Ju Tsai, M.D., Ph.D.^1,2,3,4^; Kuang-Yao Yang, M.D., Ph.D.^5,6^;
Ming-Cheng Chan, M.D., Ph.D.^7,8,9^; Kuo-Chin Kao, M.D.^10,11^;
Hao-Chien Wang, M.D., Ph.D.^12^; Wann-Cherng Perng, M.D.^13^;
Chieh-Liang Wu, M.D., Ph.D.^7,14^; Shinn-Jye Liang, M.D.^15^;
Wen-Feng Fang, M.D.^16,17^; Jong-Rung Tsai, M.D.^1,2,4^;
Wei-Ann Chang, M.D.^1,2,3^; Ying-Chun Chien, M.D.^12^;
Wei-Chih Chen, M.D.^5,18^; Han-Chung Hu, M.D. ^10,11^;
Chiung-Yu Lin, M.D.^16^; Wen-Cheng Chao, M.D., Ph.D.^7,19^;
Chau-Chyun Sheu, M.D.^1,2,3,4,*^for Taiwan Severe Influenza Research Consortium (TSIRC) Investigators

1. Division of Pulmonary and Critical Care Medicine, Department of Internal Medicine, Kaohsiung Medical University Hospital, Kaohsiung Medical University, Kaohsiung, Taiwan
2. Department of Internal Medicine, School of Medicine, College of Medicine, Kaohsiung Medical University, Kaohsiung, Taiwan
3. Graduate Institute of Clinical Medicine, College of Medicine, Kaohsiung Medical University, Kaohsiung, Taiwan
4. Department of Respiratory Therapy, College of Medicine, Kaohsiung Medical University, Kaohsiung, Taiwan
5. Department of Chest Medicine, Taipei Veterans General Hospital, Taipei, Taiwan
6. Institute of Emergency and Critical Care Medicine, School of Medicine, National Yang-Ming University, Taipei, Taiwan
7. Division of Chest Medicine, Department of Internal Medicine, Taichung Veterans General Hospital, Taichung, Taiwan
8. Central Taiwan University of Science and Technology, Taichung, Taiwan
9. Tunghai University, Taichung, Taiwan
10. Department of Thoracic Medicine, Chang Gung Memorial Hospital, Taoyuan, Taiwan
11. Department of Respiratory Therapy, Chang-Gung University College of Medicine, Taoyuan, Taiwan
12. Division of Chest Medicine, Department of Internal Medicine, National Taiwan University Hospital, Taipei, Taiwan
13. Division of Pulmonary and Critical Care Medicine, Department of Internal Medicine, Tri-Service General Hospital, National Defense Medical Center, Taipei, Taiwan
14. Center for Quality Management, Taichung Veterans General Hospital, Taichung, Taiwan
15. Division of Pulmonary and Critical Care, Department of Internal Medicine, China Medical University Hospital, Taichung, Taiwan
16. Division of Pulmonary and Critical Care Medicine, Department of Internal Medicine, Kaohsiung Chang Gung Memorial Hospital, Kaohsiung, Taiwan
17. Department of Respiratory Care, Chang Gung University of Science and Technology, Chiayi, Taiwan
18. Faculty of Medicine, School of Medicine, National Yang-Ming University, Taipei, Taiwan
19. Department of Medical Research, Taichung Veterans General Hospital, Taichung, Taiwan

* Correspondence and requests for reprints should be addressed to **Dr. Chau-Chyun Sheu**, Division of Pulmonary and Critical Care Medicine, Department of Internal Medicine, Kaohsiung Medical University Hospital, No.100, Tz-You 1st Road, 807 Kaohsiung, Taiwan, (E-mail: sheucc@gmail.com).

**Table S1. Characteristics of the study population and comparisons between survivors and non-survivors**

|  | **All Patients (N = 241)** | **Survivors (N = 174)** | **Non-survivors (N = 67)** | ***P* value** |
| --- | --- | --- | --- | --- |
| Age | 60.0 (51.0-66.0) | 60.0 (51.0-66.0) | 59.0 (51.0-67.0) | 0.610 |
| Male gender | 153 (63.5) | 111 (63.8) | 42 (62.7) | 0.882 |
| APACHE II score | 23.0 (17.0-30.0) | 21.5 (16.0-26.5) | 28.0 (23.0-34.0) | **<0.001** |
| BMI | 24.7 (21.4-28.1) | 25.0 (21.5-28.4) | 24.3 (21.4-27.2) | 0.170 |
| **Comorbidity** |  |  |  |  |
| Malignancy | 29 (12.0) | 15 (8.6) | 14 (20.9) | **0.013** |
| Diabetes | 69 (28.6) | 46 (26.4) | 23 (34.3) | 0.265 |
| Cerebrovascular disease | 18 (7.5) | 12 (6.9) | 6 (9.0) | 0.590 |
| Chronic airway disease | 21 (8.7) | 17 (9.8) | 4 (6.0) | 0.449 |
| End-stage renal disease | 14 (5.8) | 9 (5.2) | 5 (7.5) | 0.541 |
| Congestive heart failure | 26 (10.8) | 17 (9.8) | 9 (13.4) | 0.487 |
| **Influenza** |  |  |  | **0.032** |
| Type A | 179 (74.3) | 136 (78.2) | 43 (64.2) |  |
| Type B | 62 (25.7) | 38 (21.8) | 24 (35.8) |  |
| PaO_2_/FiO_2_ ratio | 84.6 (61.2-144.5) | 91.4 (64.1-160.3) | 71.6 (53.7-112.9) | **0.008** |
| **Severity of ARDS** |  |  |  | **0.032** |
| Severe ARDS | 141 (58.5) | 93 (53.4) | 48 (71.6) |  |
| Moderate ARDS | 73 (30.3) | 56 (32.2) | 17 (25.4) |  |
| Mild ARDS | 27 (11.2) | 25 (14.4) | 2 (3.0) |  |
| **Laboratory data** |  |  |  |  |
| WBC count, х10^3^/μL | 8.9 (5.9-13.6) | 8.9 (6.0-13.0) | 10.1 (5.8-16.2) | 0.239 |
| Hemoglobin, g/dL | 12.1 (10.1-13.9) | 12.4 (10.5-14.1) | 11.4 (9.5-13.5) | 0.071 |
| Platelet count, х10^3^/μL | 149.0 (106.0-204.0) | 159.5 (112.0-222.0) | 134.0 (80.0-184.0) | **0.023** |
| Albumin, g/dL | 2.9 (2.5-3.2) | 2.9 (2.6-3.3) | 2.7 (2.3-3.2) | **0.045** |
| C-reactive protein, mg/dL | 14.7 (6.2-23.1) | 14.9 (6.0-23.0) | 14.2 (6.9-24.2) | 0.554 |
| Bacterial coinfections ^*^ | 31 (12.9) | 20 (11.5) | 11 (16.4) | 0.389 |
| **Specific treatment** |  |  |  |  |
| Mechanical ventilation | 241 (100.0) | 174 (100.0) | 67 (100.0) | 1.000 |
| Prone positioning or ECMO | 92 (38.2) | 57 (32.8) | 35 (52.2) | **0.007** |
| Prone positioning | 58 (24.1) | 42 (24.1) | 16 (23.9) | 1.000 |
| ECMO | 41 (17.0) | 18 (10.3) | 23 (34.3) | **<0.001** |
| Vasopressor infusion | 121 (50.2) | 75 (43.1) | 46 (68.7) | **<0.001** |
| Hemodialysis | 40 (16.6) | 22 (12.6) | 18 (26.9) | **0.011** |

Abbreviation: APACHE II, Acute Physiology and Chronic Health Evaluation II; ARDS, acute respiratory distress syndrome; BMI, body mass index; CS, corticosteroid; ECMO, extracorporeal membrane oxygenation; FiO_2_, fraction of inspired oxygen; PaO_2_, partial pressure of arterial oxygen; WBC, white blood cell.

Statistics are presented as the median (25^th^-75^th^ percentiles) for continuous variables and as number (%) for categorical variables, as appropriate. *P* values are calculated by Mann-Whitney U test (or known as Wilcoxon Rank Sum test) and chi-square test for continuous and categorical variables, respectively.

^*^ Bacterial coinfection was defined as positive bacterial cultures from blood, pleural effusion, lower respiratory tract secretion, or urine samples within 48 hours of ARDS diagnosis.

**Table S2. Dose-dependent association between corticosteroid treatment and hospital mortality in patients with influenza-associated ARDS**

| **Cumulative dose of corticosteroid** | **Adjusted OR (95% CI) ^*^** | ***P* value** |
| --- | --- | --- |
| Within 3 days after ICU admission | 1.09 (1.02-1.16) | **0.008** |
| Within 5 days after ICU admission | 1.06 (1.02-1.11) | **0.007** |
| Within 7 days after ICU admission | 1.04 (1.01-1.08) | **0.018** |
| Within 10 days after ICU admission | 1.02 (0.99-1.04) | 0.207 |

Abbreviation: APACHE II, Acute Physiology and Chronic Health Evaluation II; ARDS, acute respiratory distress syndrome; CI, confidence interval; ECMO, extracorporeal membrane oxygenation; ICU, intensive care unit; OR, odds ratio.

**^*^** The adjusted OR (95% CI) of hospital mortality per 100 mg increment of hydrocortisone equivalent dose were calculated using the logistic regression model adjusting for APACHE II score, underlying malignancy, influenza type, and ECMO treatment.

**Table S3. Association between corticosteroid treatment and hospital mortality in patients with influenza-associated ARDS, according to the timing of corticosteroid treatment**

| **Timing of CS treatment ^*^** | **Survivors (N = 174)** | **Non-survivors (N = 67)** | ***P*  value ^†^** | **Adjusted OR (95% CI) ^‡^** | ***P*  value ^c^** |
| --- | --- | --- | --- | --- | --- |
| No corticosteroid treatment | 82 | 20 | **<0.001** | ref |  |
| ICU Day 1-3 | 48 | 37 |  | 4.58 (2.06,10.17) | **<0.001** |
| ICU Day 4-7 | 18 | 3 |  | 0.57 (0.12,2.77) | 0.192 |
| ICU Day 8-14 | 26 | 7 |  | 0.87 (0.27,2.76) | 0.440 |

Abbreviation: APACHE II, Acute Physiology and Chronic Health Evaluation II; ARDS, acute respiratory distress syndrome; CI, confidence interval; ECMO, extracorporeal membrane oxygenation; ICU, intensive care unit; OR, odds ratio.

**^*^** The timing of CS treatment was defined as the day reaching cumulative hydrocortisone equivalent dose ≥ 200 mg.

^†^ χ^2^ test

**^‡^** The adjusted OR and 95% CI of hospital mortality were calculated using multivariable logistic regression models adjusting for APACHE II scores, underlying malignancy, influenza type, and ECMO treatment.

**Table S4. Associations between early corticosteroid treatment and subsequent bacterial infections in patients with influenza-associated ARDS**

| **Positive culture after Day 3** | **No early CS treatment** | **Early CS treatment** | ***P* value** |
| --- | --- | --- | --- |
| **In any samples** |  |  |  |
| Yes (N = 92) | 55 | 37 | 0.203 ^†^ |
| No (N = 129) | 88 | 41 |  |
| **OR (95% CI) ^*^** | ref | 1.67 (0.94-2.99) | 0.082 |
|  |  |  |  |
| **In respiratory samples** |  |  |  |
| Yes (N = 77) | 48 | 29 | 0.658 ^†^ |
| No (N = 144) | 95 | 49 |  |
| **OR (95% CI) ^*^** | ref | 1.36 (0.75-2.49) | 0.316 |
|  |  |  |  |
| **In blood samples** |  |  |  |
| Yes (N = 27) | 13 | 14 | 0.084 ^†^ |
| No (N = 194) | 130 | 64 |  |
| **OR (95% CI) ^*^** | ref | 2.37 (1.01-5.56) | 0.048 |
|  |  |  |  |
| **In urine samples** |  |  |  |
| Yes (N = 18) | 12 | 6 | 1.000 ^†^ |
| No (N = 203) | 131 | 72 |  |
| **OR (95% CI) ^*^** | ref | 0.99 (0.35-2.81) | 0.984 |

Abbreviation: ARDS, acute respiratory distress syndrome; CI, confidence interval; CS, corticosteroid; OR, odds ratio.

^*^ The OR and 95% CI of developing subsequent bacterial infections in patients with early corticosteroid treatment, compared to those without early corticosteroid treatment, were calculated using univariate logistic regression models.

^†^ χ^2^ test

**Table S5. Associations between subsequent bacterial infections and hospital mortality in patients with influenza-associated ARDS**

| **Positive culture after Day 3** | **Survivors (N = 158)** | **Non-survivors (N = 63)** | ***P*  value ^*^** | **Adjusted OR (95% CI) ^†^** | ***P*  value ^†^** |
| --- | --- | --- | --- | --- | --- |
| In any samples | 58 | 34 | **0.023** | 1.50 (0.70-3.19) | 0.298 |
| In respiratory samples | 45 | 32 | **0.003** | 2.20 (1.01-4.81) | **0.048** |
| In blood samples | 17 | 10 | 0.362 | 0.73 (0.24-2.20) | 0.573 |
| In urine samples | 13 | 5 | 1.000 | 1.39 (0.40-4.81) | 0.599 |

Abbreviation: APACHE II, Acute Physiology and Chronic Health Evaluation II; ARDS, acute respiratory distress syndrome; CI, confidence interval; ECMO, extracorporeal membrane oxygenation; OR, odds ratio.

^*^ χ^2^ test

**^†^** The adjusted OR and 95% CI of hospital mortality were calculated using multivariable logistic regression models adjusting for APACHE II scores, underlying malignancy, influenza type, and ECMO treatment.

**Table S6. Start day, duration, and total dose of corticosteroid treatment between survivors and non-survivors in patients with influenza-associated ARDS who ever received corticosteroid treatment within 14 days after ICU admission**

| **Ever received CS treatment at any dose within 14 days after ICU admission** | **Survivors (N = 104)** | **Non-survivors (N = 50)** | ***P* value** |
| --- | --- | --- | --- |
| **Interval between ICU admission and the start of CS treatment ^*^, days** | 1.0 (0.0-7.0) | 0.5 (0.0-2.0) | **0.036** |
| ≤ 3 days | 66 (63.5) | 42 (84.0) | **0.029** |
| 3-7 days | 14 (13.5) | 2 (4.0) |  |
| 8-14 days | 24 (23.1) | 6 (12.0) |  |
|  |  |  |  |
| **Duration of CS treatment ^†^, days** | 13.0 (7.0-14.0) | 13.5 (12.0-14.0) | 0.054 |
|  |  |  |  |
| **Total dose of CS treatment by 14 days ^‡^, 100mg hydrocortisone equivalent dose** | 11.6 (7.0-20.0) | 11.5 (7.5-20.2) | 0.965 |

Abbreviation: ARDS, acute respiratory distress syndrome; CI, confidence interval; CS, corticosteroid; ICU, intensive care unit; OR, odds ratio.

Data are presented as the median (25^th^-75^th^ percentiles). *P* values are calculated by Mann-Whitney U test.

^*^ If a patient received CS treatment on the first ICU day, then the interval was calculated as zero day.

^†^ Duration is calculated as the total day that a patient receiving CS treatment during the first 14 days in ICU, regardless of dose.

^‡^ Total dose of CS was presented as 100 mg hydrocortisone equivalent dose

**Table S7. Characteristics and outcomes between patients with versus without early corticosteroid treatment in the propensity score-matched cohort of influenza-associated ARDS**

|  | **Early CS Treatment (N = 85)** | **No Early CS Treatment (N = 85)** | ***P* value** |
| --- | --- | --- | --- |
| Age | 61.0 (56.0-68.0) | 60.0 (48.0-67.0) | 0.432 |
| Male gender | 52 (61.2) | 60 (70.6) | 0.257 |
| APACHE II score | 24.0 (18.0-30.0) | 25.0 (18.0-30.0) | 0.562 |
| BMI | 24.6 (22.1-28.1) | 25.4 (22.8-28.1) | 0.868 |
| **Comorbidity** |  |  |  |
| Malignancy | 7 (8.2) | 14 (16.5) | 0.160 |
| Diabetes | 26 (30.6) | 27 (31.8) | 1.000 |
| Cerebrovascular disease | 8 (9.4) | 8 (9.4) | 1.000 |
| Chronic airway disease | 10 (11.8) | 9 (10.6) | 1.000 |
| End-stage renal disease | 7 (8.2) | 5 (5.9) | 0.766 |
| Congestive heart failure | 11 (12.9) | 11 (12.9) | 1.000 |
| **Influenza** |  |  | 0.617 |
| Type A | 61 (71.8) | 57 (67.1) |  |
| Type B | 24 (28.2) | 28 (32.9) |  |
| PaO_2_/FiO_2_ ratio | 87.5 (56.0-142.3) | 80.1 (61.5-136.3) | 0.924 |
| **Severity of ARDS** |  |  | 0.617 |
| Severe ARDS | 46 (54.1) | 51 (60.0) |  |
| Moderate ARDS | 32 (37.6) | 25 (29.4) |  |
| Mild ARDS | 7 (8.2) | 9 (10.6) |  |
| **Laboratory data** |  |  |  |
| WBC count, х10^3^/μL | 9.6 (5.5-15.5) | 9.3 (6.5-13.6) | 0.709 |
| Hemoglobin, g/dL | 12.5 (10.5-14.2) | 11.9 (10.2-13.9) | 0.309 |
| Platelet count, х10^3^/μL | 150.0 (108.5-200.5) | 147.0 (92.0-206.0) | 0.952 |
| Albumin, g/dL | 2.9 (2.5-3.3) | 2.8 (2.5-3.2) | 0.774 |
| C-reactive protein, mg/dL | 14.1 (4.2-23.3) | 13.4 (5.5-21.2) | 0.624 |
| Bacterial coinfections ^*^ | 14 (16.5) | 11 (12.9) | 0.665 |
| **Specific treatment** |  |  |  |
| Mechanical ventilation | 85 (100.0) | 85 (100.0) | 0.509 |
| Prone positioning | 29 (34.1) | 21 (24.7) | 0.238 |
| ECMO | 11 (12.9) | 16 (18.8) | 0.401 |
| Vasopressor infusion | 49 (57.6) | 42 (49.4) | 0.356 |
| Hemodialysis | 16 (18.8) | 17 (20.0) | 1.000 |
| **Clinical outcomes** |  |  |  |
| Hospital mortality, n (%) | 37 (43.5) | 18 (21.2) | **0.003** |
| Hospital days in survivors, day | 24.8 (17.1-40.1) | 28.1 (21.5-47.1) | 0.190 |
| ICU days in survivors, day | 13.8 (8.4-23.0) | 17.2 (9.9-21.8) | 0.390 |
| Ventilator days in survivors, day | 11.9 (8.4-25.3) | 16.4 (8.4-22.3) | 0.547 |
| ICU-free days at Day 28 | 0.0 (0.0-15.2) | 8.0 (0.0-17.6) | 0.088 |
| Ventilator-free days at Day 28 | 0.0 (0.0-17.1) | 9.7 (0.0-18.5) | 0.080 |

Abbreviation: APACHE II, Acute Physiology and Chronic Health Evaluation II; ARDS, acute respiratory distress syndrome; BMI, body mass index; CS, corticosteroid; ECMO, extracorporeal membrane oxygenation; FiO_2_, fraction of inspired oxygen; PaO_2_, partial pressure of arterial oxygen; WBC, white blood cell.

To account for residual confounding by indication of the associations between early corticosteroid treatment and clinical outcomes, variables potentially associated with early corticosteroid treatment, including age, sex, APACHE II score, influenza type, ARDS severity, bacterial coinfection, vasopressor infusion, prone positioning, ECMO treatment, and chronic airway disease, were included in a logistic regression model with early corticosteroid treatment as the dependent variable to determine a propensity score for treatment. A propensity score-matched cohort was then selected from the original cohort.

Statistics are presented as the median (25^th^-75^th^ percentiles) for continuous variables and as number (%) for categorical variables, as appropriate. *P* values are calculated by Mann-Whitney U test (or known as Wilcoxon Rank Sum test) and chi-square test for continuous and categorical variables, respectively.

^*^ Bacterial coinfection was defined as positive bacterial cultures from blood, pleural effusion, lower respiratory tract secretion, or urine samples within 48 hours of ARDS diagnosis.

**Table S8. Univariate and multivariable analyses of factors associated with hospital mortality in the propensity score-matched cohort of influenza-associated ARDS**

|  | **Univariate Analysis ^*^** | | **Multivariable Analysis ^†^** | |
| --- | --- | --- | --- | --- |
|  | **OR (95% CI)** | ***P* value** | **Adjusted OR (95% CI)** | ***P* value** |
| APACHE II score | 1.10 (1.05-1.15) | **<0.001** | 1.10 (1.05-1.16) | **<0.001** |
| PaO_2_/FiO_2_ ratio | 0.99 (0.99-1.00) | **0.014** |  |  |
| WBC | 1.00 (1.00-1.00) | **0.025** |  |  |
| Platelet | 0.99 (0.99-1.00) | **0.022** |  |  |
| Vasopressor infusion | 2.32 (1.19-4.55) | **0.014** |  |  |
| ECMO | 5.73 (2.37-13.9) | **0.000** | 9.10 (3.18-26.0) | **<0.001** |
| Hemodialysis | 2.37 (1.09-5.14) | **0.030** |  |  |
| **Early CS treatment** | 2.87 (1.46-5.63) | **0.002** | 4.30 (1.90-9.75) | **0.001** |

Abbreviation: APACHE II, Acute Physiology and Chronic Health Evaluation II; ARDS, acute respiratory distress syndrome; BMI, body mass index; CI, confidence interval; CS, corticosteroid; ECMO, extracorporeal membrane oxygenation; FiO_2_, fraction of inspired oxygen; PaO_2_, partial pressure of arterial oxygen; OR, odds ratio; WBC, white blood cell.

**^*^** The variable representing early CS treatment, basic demographic variables, and all clinical variables possibly associated with hospital mortality were analyzed in univariate logistic regression models.

**^†^** Variables associated with hospital mortality with a *p* value < 0.05 in univariate models were selected into the multivariable logistic regression model, using a stepwise algorithm with criteria of *p* > 0.05 for eliminating variables.

**Figure S1. Enrollment and follow-up of the study population**


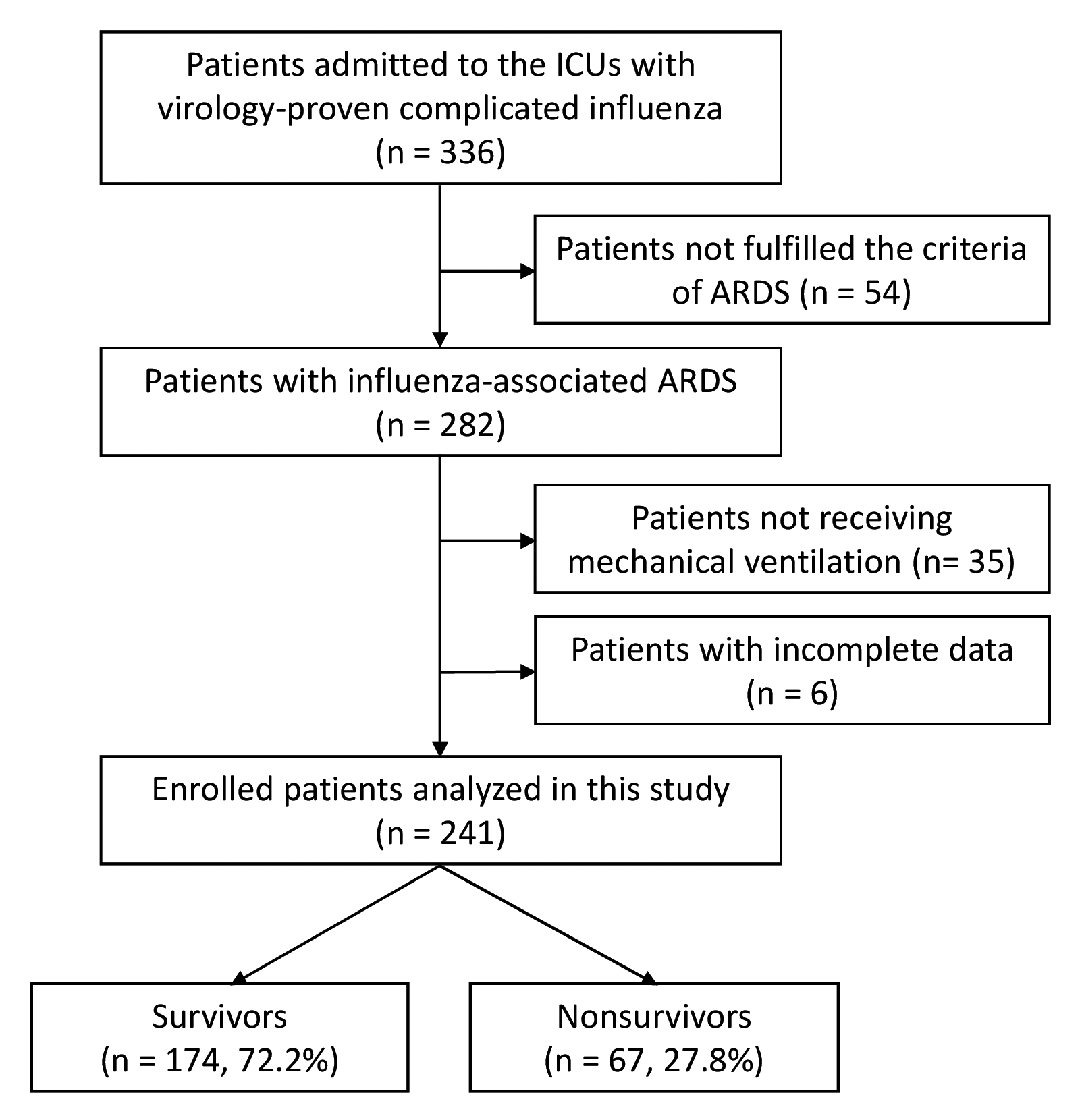


**Figure S2. Cumulative proportion of influenza-associated ARDS patients receiving corticosteroid treatment of ≥ 200 mg hydrocortisone equivalent dose**


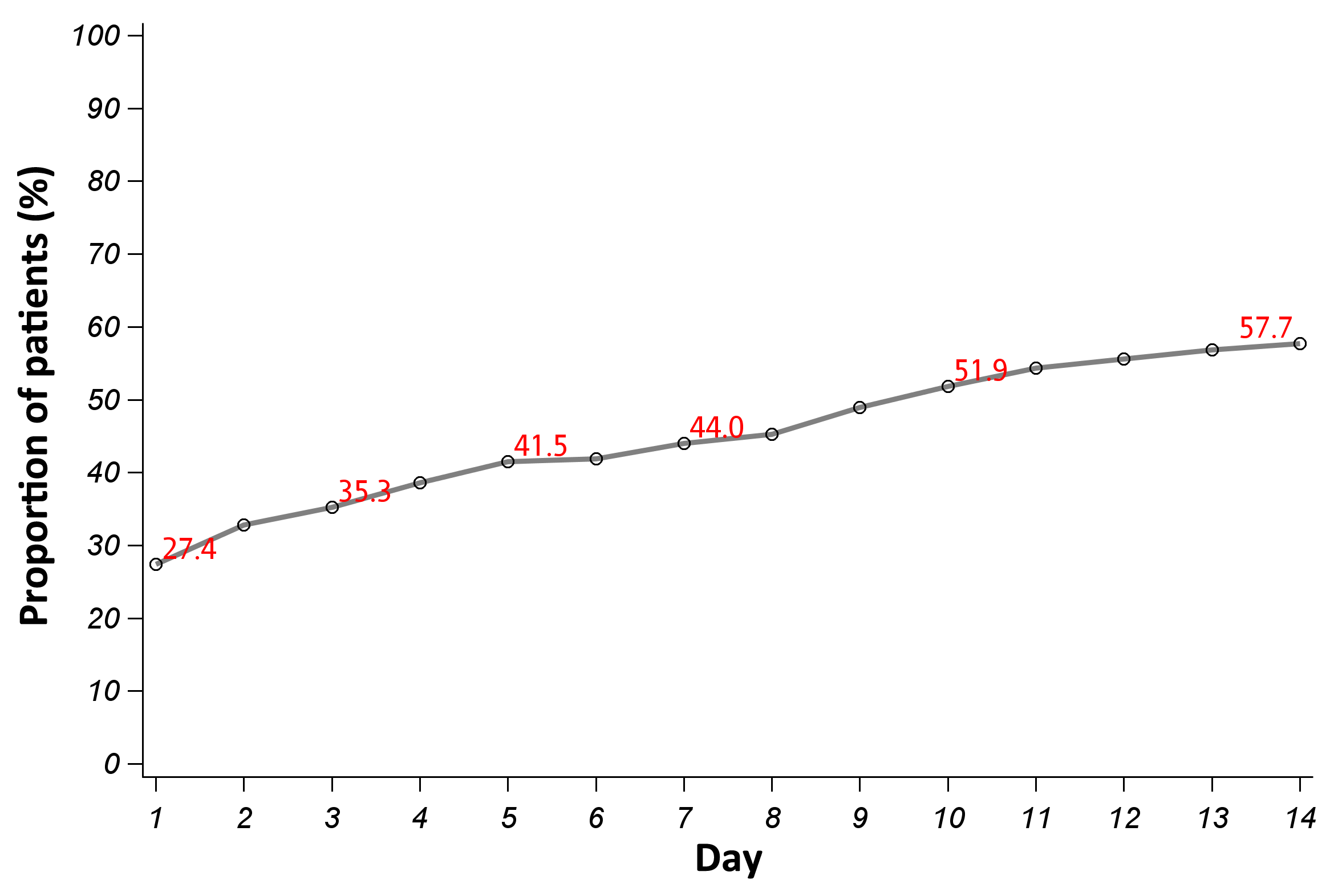


**Figure S3. Probability of survival from ICU admission to Day 30 in the propensity score-matched cohort of influenza-associated ARDS, according to whether patients received early corticosteroid (CS) treatment or not**


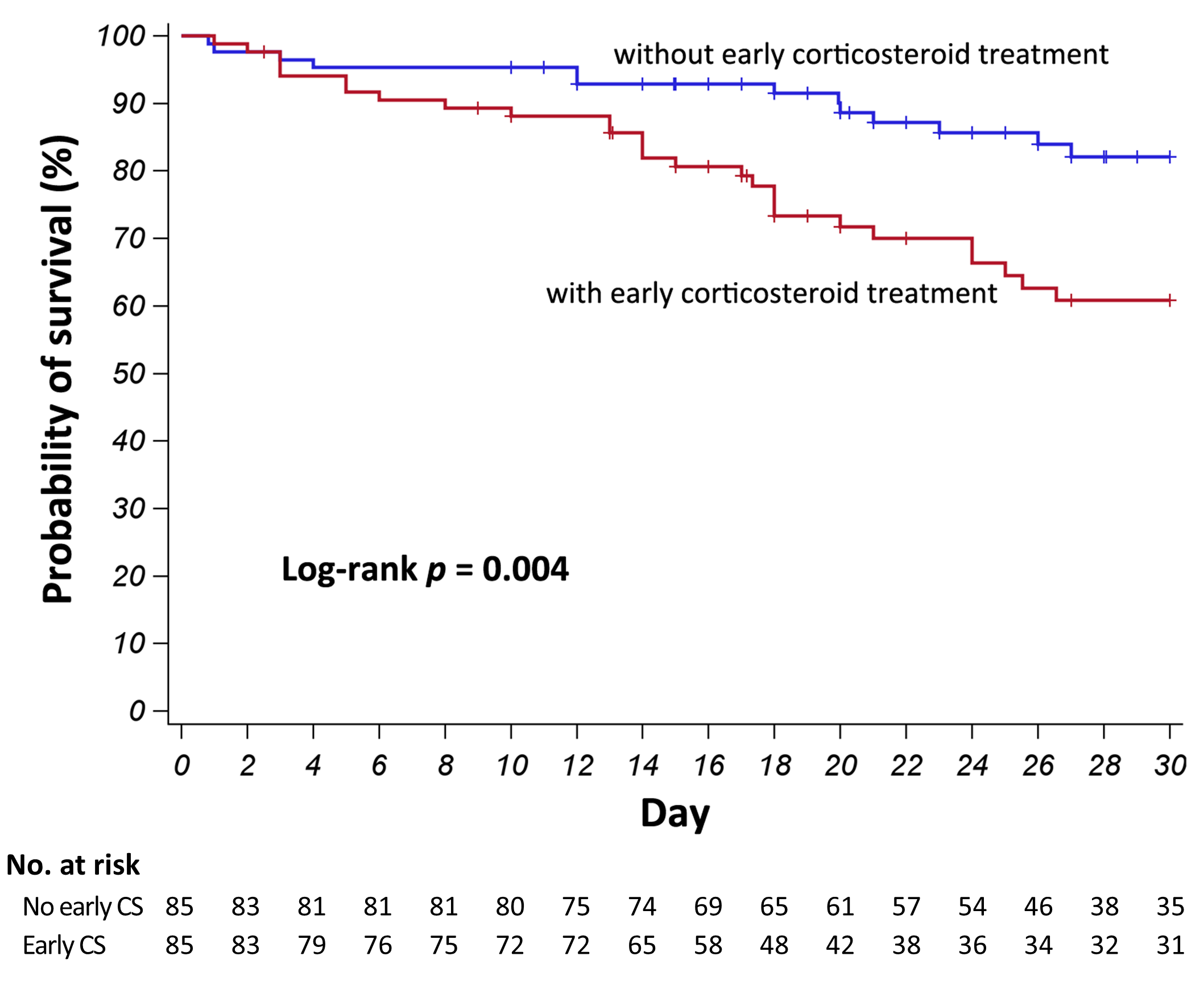


**Figure S4. Forest Plot of adjusted odds ratio of hospital mortality in the propensity score-matched cohort of influenza-associated ARDS.**Odds ratios were adjusted for APACHE II score and ECMO treatment, which were selected from the multivariable model in Table S8.


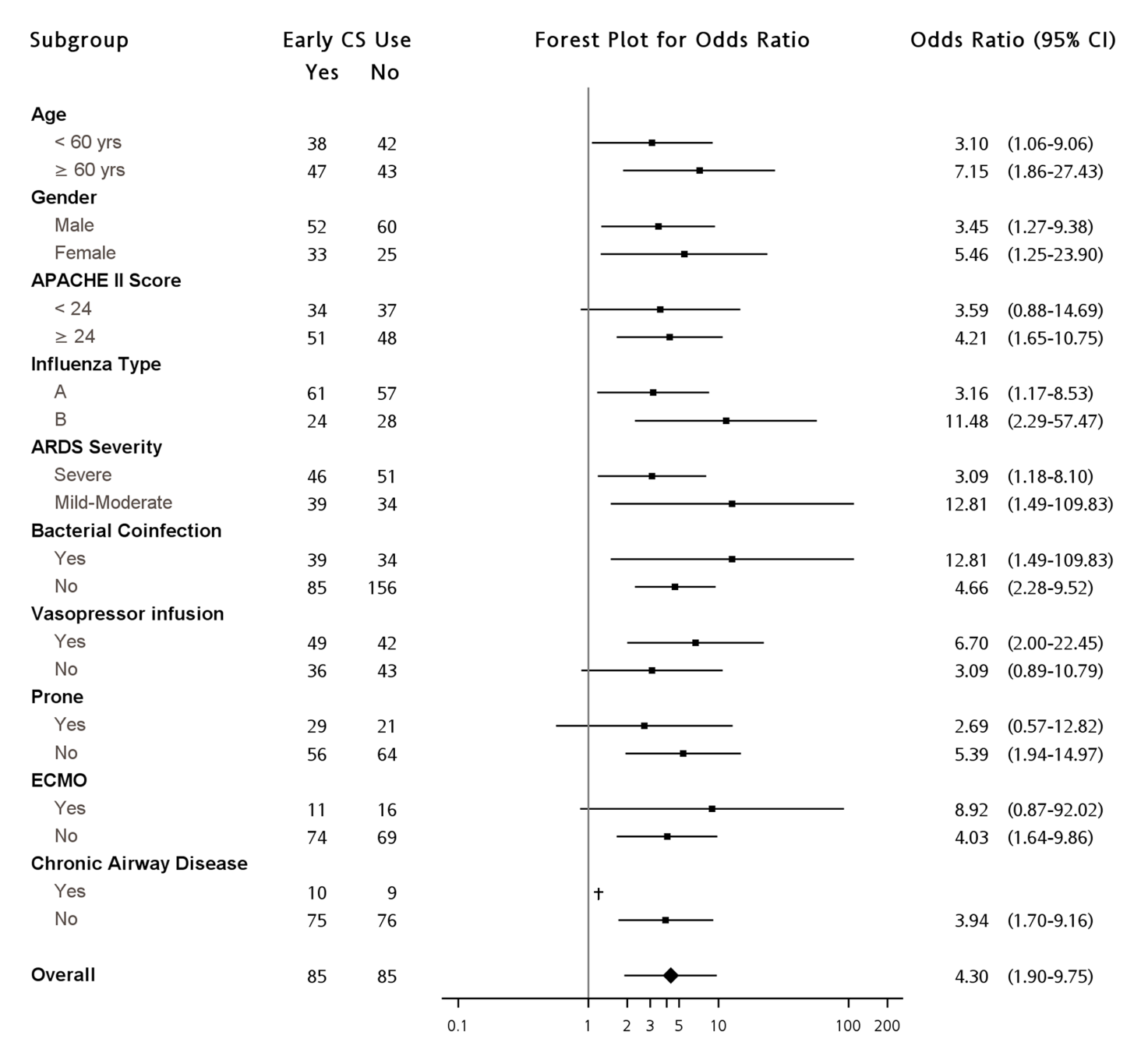


^†^ The adjusted OR could not be calculated due to small sample size.
